# A rare case of pediatric autoimmune pancreatitis and autoimmune hepatitis in a patient with sickle cell disease

**DOI:** 10.1002/jpr3.70100

**Published:** 2025-10-21

**Authors:** Sasha‐Jane Abi‐Aad, Michelle Edward, David Germain, Rene Gomez‐Esquivel, Racha Khalaf

**Affiliations:** ^1^ Department of Pediatrics, Division of Pediatric Gastroenterology, Hepatology and Nutrition University of South Florida Morsani College of Medicine Tampa Florida USA; ^2^ Department of Internal Medicine/Pediatrics University of South Florida Morsani College of Medicine Tampa Florida USA; ^3^ Department of Radiology University of South Florida Morsani College of Medicine Tampa Florida USA

**Keywords:** hepatopancreatitis, IgG4‐related disease, pediatric autoimmune disease

## Abstract

Concurrent pediatric autoimmune pancreatitis (AIP) and autoimmune hepatitis (AIH) are rarely reported, and no established pediatric‐specific guidelines are available to guide the diagnosis and management of these conditions in children. While AIP and AIH share an underlying autoimmune mechanism of injury, marked by chronic inflammatory changes such as fibrosis, sclerosis, and infiltration of mononuclear cells like plasma cells and lymphocytes, their concurrent occurrence is infrequent, as they remain distinct clinical entities. In this case report, we present a case of concurrent AIH and AIP with treatment challenges secondary to underlying sickle cell disease (SCD) in an 11‐year‐old female. Herein, we discuss not only the diagnostic challenges faced when determining the cause of cholestasis in a patient with SCD but also the therapeutic strategies adopted throughout the care of this patient.

## 1 INTRODUCTION

Pediatric autoimmune pancreatitis (P‐AIP) is rare, with a prevalence of 3.9% in the INSPPIRE cohort.^1^ There are two distinct subtypes. Type 1 is an immunoglobulin (Ig) G4 related disease (IgG4‐RD), a systemic disease typically seen in older individuals and characterized by elevated serum IgG4, periductal lymphocytes infiltration, and abundant IgG4 plasma cells, and type 2 is more common in younger patients and marked by ductal lymphocytic infiltration and fibrosis with minimal IgG4 plasma cells.^2^ While adult‐AIP and IgG4‐RD require a combination of radiological, laboratory, and histopathological criteria, diagnosing P‐AIP is more challenging as manifestations may differ and subtypes can overlap.^3^ Pediatric autoimmune hepatitis (AIH) is also a rare disease with a prevalence of 2.4–9.9 per 100,000.^4^ We report an uncommon case of concurrent pediatric AIP and AIH with elevated serum IgG4 but negative tissue immunostaining, in an 11‐year‐old female with sickle cell disease (SCD).

## 2 CASE REPORT

An 11‐year‐old female with SCD (HbSS) and recurrent acute pain crises was seen in the emergency department (ED)for jaundice, epigastric, and chest pain. Five days earlier, during a routine follow‐up, she had abnormal liver enzymes, hyperbilirubinemia, and mild scleral icterus despite a normal review of systems and was advised to hold hydroxyurea for 2 weeks. She returned with severe abdominal pain. On examination, she was tachycardic with scleral icterus and right upper quadrant tenderness; the remainder of the physical exam was unremarkable. Labs showed normocytic normochromic anemia, worsened transaminitis, and conjugated hyperbilirubinemia. Lipase was normal (Table 1).

**Table 1:** Laboratory results from preadmission to postdischarge in an 11‐year‐old girl with SCD presenting to the ED with abdominal pain and jaundice.

	Scheduled follow‐up visit	At admission (ED)	First visit after discharge (1 month later)	50 days after discharge	Reference values
Days from first visit	Day 0	Day 5	Day 31	Day 68	
Laboratory tests					
Hemoglobin	7.6	7.9	8.4	8.4	11.5–15.5 g/dL
MCV	87.4	80.5	88.6	85.9	77.0–95.0 fL
MCH	29.1	29.6	30.0	30.4	27.0–31.2 pg
White blood cells	14	14.2	14.2	14.2	6.0–17.5 $\times 10^{3}$/μ L
ESR	‐	67	‐	‐	0–20 mm/h
CRP	‐	‐	6.3	‐	<8.0 mg/L
High sensitivity CRP	‐	7.4	‐	‐	0.01–0.5 mg/dL
Platelets	590	545	510	323	142.0–424.0 $\times 10^{3}$/μ L
AST	294	631	63	192	5–34 U/L
ALT	131	239	37	141	5–55 U/L
GGT	‐	22	39	51	9–36 U/L
Total bilirubin	8.2	16.1	5.3	6.5	0.1–1.2 mg/dL
Direct bilirubin	‐	10.3	1.9	2.7	0.1–0.5 mg/dL
ALP	247	351	248	371	141–460 U/L
Albumin	3.5	2.8	3.7	3.5	3.5–5.0 g/dL
Total protein	9.2	9.4	8.2	9.2	6–8 g/dL
Prothrombin time	‐	17.7	11.0	14.3	10.0–14.4 s
INR	‐	1.4	1.1	1.2	
Lipase	‐	21	‐	‐	4–78 U/L
**Anthropometrics**					
Weight (kg)	38.6	38.6	39.4	43.1	
Height (cm)	152.3	152.3	153.6	154.5	
BMI $({\mathrm{kg}/m}^{2}$)	16.6	16.6	16.7	18.1	

Abbreviations: ALP, alkaline phosphatase; ALT, alanine aminotransferase; AST, aspartate aminotransferase; BMI, body mass index; CRP, C‐reactive protein; ED, emergency department; ESR, erythrocyte sedimentation rate; GGT, gamma‐glutamyl transferase; INR, international normalized ratio; MCH, mean corpuscular haemoglobin; MCV, mean corpuscular concentration; SCD, sickle cell disease.

**Figure 1:**
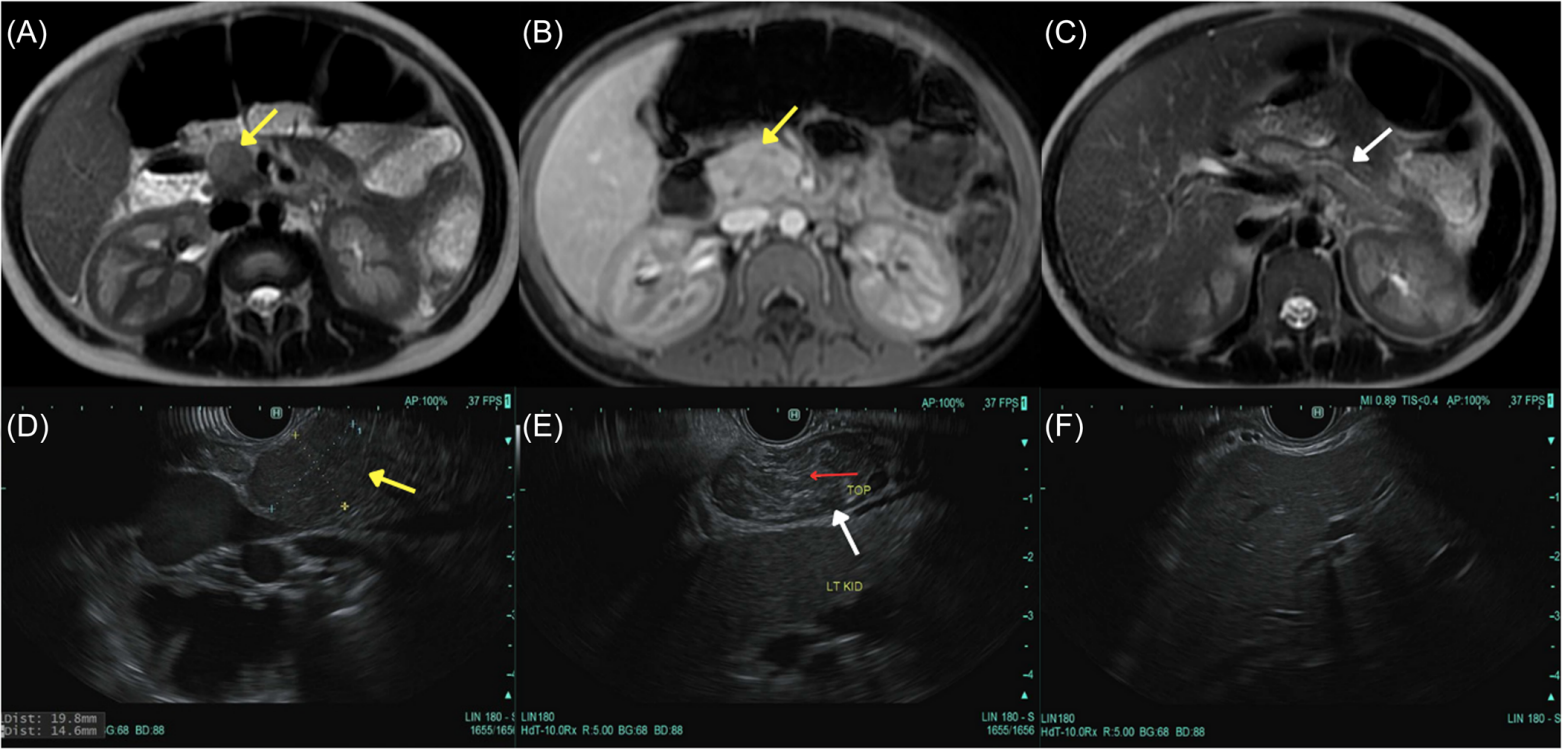
(A) T2 axial MRI section, demonstrating focal mass‐like area (yellow arrow) of high T2 signal in pancreatic head and uncinate process measuring 2.1 ×1.8 cm with no significant associated bile duct or pancreatic ductal distention. There is a thin peripheral hypointense capsule‐like rim on T2 imaging. (B) T1 axial MRI section with gadolinium contrast demonstrating homogenous enhancement similar to normal pancreas throughout the mass like area (yellow arrow). (C) T2 axial MRI section, showing downstream edema throughout the body and tail (white arrow) of the pancreas. (D) EUS image showing an oval, isoechoic and homogenous mass (yellow arrow) at the uncinate of the pancreas, measuring 19 mm in maximal cross‐sectional diameter. (E) EUS image showing the tail (Top‐ white arrow) of the pancreas with normal caliber pancreatic duct (red arrow). The echogenicity is slighly heterogenous described as “salt pepper” with some hyperechoigenic strands which the apperance is considered normal for the patient's age. (F) EUS image showing homogenous parenchyma, with no sign of significant endosonographic abnormality in the left or right lobes of the liver. No focal pathology or masses were identified. This image only shows the left lobe of the liver. There was no sign of significant endosonographic abnormality in the common bile duct. The maximum diameter of the duct was 3 mm. Ducts of normal caliber and with regular contour were identified. No masses were seen. EUS, endoscopic ultrasound; MRI, magnetic resonance imaging.

**Figure 2:**
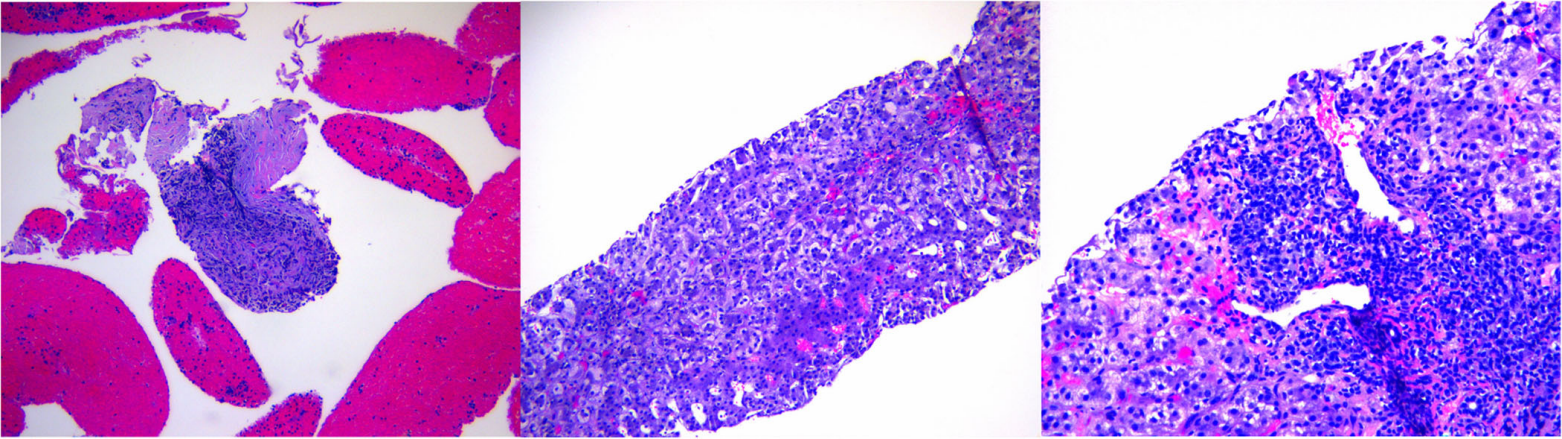
Histopathology photomicrograph stained of an 11‐year‐old girl with sickle cell disease, jaundice and a pancreatic mass. (A) H&E section of the pancreatic mass showing residual pancreatic tissue with fibrosis and chronic inflammation (magnified ×100). (B) H&E section from the liver showing cholestatic hepatitis with extensive feathery degeneration (magnified ×200). (C) Section from the portal tract showing lymphoplasmacytic infiltration with increased plasma cells and bile ductular proliferation (magnified ×200). H&E, hematoxylin and eosin.

Workup was ordered (Supporting Information S1). Magnetic resonance cholangiopancreatography (MRCP) revealed a 2.3 cm mass at the uncinate process with extensive abnormal pancreatic signal (Figure 1A–C), and mild heterogenous liver enhancement. Endoscopic ultrasound (EUS) revealed an isoechoic, homogenous mass with diffuse echogenicity and hyperechoic strands in the body and tail (Figure 1D–G). Fine needle aspiration of the mass revealed chronic inflammation (Figure 2A). Liver biopsy showed cholestatic hepatitis with mild interface hepatitis, and increased plasma cells (Figure 2B,C). Immunostaining was negative for IgG4+ plasma cells in both tissues; however, antismooth muscle antibody (SMA) was 75 (reference <20 U), total serum IgG 3514 (reference 480–1530 mg/dL) and serum IgG4 of 282.4 (reference 2.0–115.0 mg/dL) (Supporting Information S1). She was diagnosed with both AIP and AIH and discharged on prednisone 40 mg following a 14‐day admission.

Two days after discharge, she experienced an acute pain crisis attributed to prednisone and was switched to 9 mg of budesonide. At follow‐up, liver enzymes improved, pancreatic function normalized (elastase 469 mcg/g), and azathioprine 150 mg was initiated alongside budesonide. Fifty days later, aspartate aminotransferase and alanine aminotransferase worsened due to poor compliance, reflected by diminished 6‐thioguanine levels (Table 1); however, MRCP revealed a stable pancreatic mass with reduced signals. Following motivational interviewing, liver enzymes and hepatic synthetic function improved, and budesonide tapering began after 5 months. By 9 months, she was completely off budesonide, and MRCP demonstrated resolution of the mass. She remains on azathioprine and has been in remission for >20 months.

## 3 DISCUSSION

This is a rare case of concurrent AIH and AIP in a pediatric patient with SCD. The patient met INSPPIRE diagnostic criteria for P‐AIP, presenting with abdominal pain, a pancreatic mass, focal enlargement with hypointense capsule‐like rim on T2 imaging, dense lymphocytic infiltrates, and serum IgG4 > 2x the upper limit normal (ULN), despite normal lipase and absent IgG4 immunostaining. According to INSPPIRE, P‐AIP diagnosis can be based solely on clinical and imaging findings. Pancreatic enzymes may be normal in up to 57% of cases, and EUS‐biopsy is often not feasible in children. Additionally, P‐AIP may have overlapping features of both subtypes, and subtype differentiation is more challenging in pediatric patients, with elevated serum IgG4 reported in only 22% of cases.^3^ She had elevated serum IgG4 consistent with type 1 AIP, but lacked IgG4 plasma cells on immunostaining, more characteristic of type 2. Possible explanations are inadequate tissue sampling, early disease, or overlapping features, previously reported in pediatric cases. Concurrent AIH was diagnosed using simplified International Autoimmune Hepatitis Group (IAIHG) criteria: elevated total serum IgG (>1.10 x ULN), positive anti‐SMA, histological evidence of active hepatitis, and exclusion of other causes.^5^

It is unclear whether AIP or AIH developed first; however, their concurrent presentation with elevated serum IgG4 raises the possibility of IgG4‐RD involving both the pancreas and liver. According to the 2020 revised IgG4‐RD criteria, a diagnosis is possible when clinical or radiologic features show a mass, or diffuse or localized swelling, with serum IgG4 > 135 mg/dL. However, a definite diagnosis requires characteristic histopathology.^6^ The patient had a pancreatic mass with serum IgG4 of 282.4 mg/dL, making IgG4‐RD a possible differential. In addition, hepatic involvement may be categorized as either IgG4‐related AIH or IgG4‐related hepatopathy. While there's no single definition for IgG4 AIH, most agree that it requires meeting the established criteria for classical AIH, with IgG4 plasma cell infiltration. In contrast, IgG4‐related hepatopathy refers to liver and bile duct injury in the context of IgG4‐RD, without meeting AIH criteria.^7^ The patient met AIH criteria and had high serum IgG4 but lacked IgG4 immunostaining in the liver.

The simultaneous occurrence of AIP and AIH in the absence of prior autoimmune disease, along with high serum IgG4, suggests a possible uncharacterized variant of IgG4‐AIP and AIH. Most IgG4‐RD occur in adults, with few pediatric reports, which limit our understanding of how it manifests in children.

The presence of SCD introduced additional diagnostic and management challenges. Liver injury is not uncommon in SCD, and SCD hepatopathy includes acute and chronic manifestations, such as acute sickle cell hepatic crisis, intrahepatic cholestasis, cholelithiasis, iron overload, or drug‐induced liver injury.^8^ She had stable normocytic anemia, indicating no acute sickle cell crisis. The absence of sickling or iron deposition on histology, but the presence of lymphocytic infiltration, makes SCD hepatopathy unlikely and supports the diagnosis of autoimmune disease. The patient did not tolerate prednisone due to worsened pain crises and was transitioned to budesonide, a second‐line therapy for AIP and AIH. A meta‐analysis reported that systemic corticosteroids increase hospital readmission risk due to pain crises in SCD^9^; however, another study found no such risk with inhaled corticosteroids,^10^ indicating potential safe use of budesonide in this population.

This case highlights the efficacy of other steroids like budesonide in managing autoimmune disorders in pediatric patients intolerant to prednisone. Future research should investigate the role of SCD in early‐onset autoimmune disorders and establish management guidelines specific to this population.

## 4 CONCLUSION

Considering these diagnostic challenges, our case highlights the need for revised pediatric IgG4‐RD criteria and multicenter studies to better define the disease in children, and provides direction for prognostic factors and disease severity scales in pediatric populations.

## CONFLICT OF INTEREST STATEMENT

The authors declare no conflicts of interest.

## ETHICS STATEMENT

Written informed consent was obtained from the parents to share this case.

## Supporting information

Supporting information.
