# Supplementary material for: A rare case of pediatric autoimmune pancreatitis and autoimmune hepatitis in a patient with sickle cell disease
Source: JPGN Rep. 2025 Oct 21;7(1):142–6. doi: 10.1002/jpr3.70100 (PMC12894056; doi:10.1002/jpr3.70100)
Supplement: Supplementary file 1 — Supporting information. [file JPR3-7-142-s001.docx]

**Supplemental Material**

**Title : Clinical Presentation and Additional Diagnostic Investigations**

The patient did not experience any other gastrointestinal symptoms or signs of encephalopathy, and the remainder of the physical exam was normal. Her vital signs on admission were notable for a heart rate of 122 beats per minute, blood pressure of 120/72 mmHg, and normal oxygen saturation and temperature.

After a thorough examination, an abdominal ultrasound was performed, revealing cholelithiasis without active inflammation, with normal-appearing liver and pancreas. Workup for viral hepatitis (hepatitis A, hepatitis B, hepatitis C, hepatitis E, Epstein-Barr virus, and cytomegalovirus), acetaminophen toxicity, α-1 antitrypsin deficiency, and Wilson disease was negative.

Following magnetic resonance cholangiopancreatography, endoscopic ultrasound, and biopsy, an autoimmune workup was ordered. Serum IgG and IgG4 were elevated, and anti-smooth muscle antibody (SMA) was positive; however, antinuclear antibody (ANA), liver kidney microsomal antibody (LKM), and mitochondrial antibody were negative.

She was found to have exocrine pancreatic insufficiency (fecal elastase of 22 mcg/g), and severe vitamin D and A deficiencies, and started on pancrealipase and multivitamins. Endocrine function was normal with a hemoglobin A1c (HbA1c) of 4.3 %.
